# 903. Prevalence of Underlying Conditions Associated With Higher Risk for Severe RSV, Influenza, or COVID-19 in Adults in the United States, 2017-2018

**DOI:** 10.1093/ofid/ofad500.948

**Published:** 2023-11-27

**Authors:** Parinaz Ghaswalla, Abby Hitchens, Sean D Candrilli, Justin Carrico, Katherine A Hicks, Eleanor Wilson, Darshan Mehta, Catherine A Panozzo

**Affiliations:** Moderna, Inc., Cambridge, Massachusetts; RTI Health Solutions, Research Triangle Park, North Carolina; RTI Health Solutions, Research Triangle Park, North Carolina; RTI Health Solutions, Research Triangle Park, North Carolina; RTI Health Solutions, Research Triangle Park, North Carolina; Moderna, Inc., Cambridge, Massachusetts; Moderna, Inc., Cambridge, Massachusetts; Moderna, Inc., Cambridge, Massachusetts

## Abstract

**Background:**

Respiratory syncytial virus (RSV), influenza, and COVID-19 pose a significant health risk to older adults and individuals with certain underlying health conditions. The descriptive epidemiology of such conditions can guide efforts in development of interventional strategies. The objective of this study was to estimate the prevalence in the United States (US) of underlying health conditions by age and race/ethnicity that are associated with high risk for developing severe RSV, influenza, or COVID-19.

**Methods:**

This study analyzed data from medical assessments and self-reported demographic, socioeconomic, and health-related information from the 2017-2018 National Health and Nutrition Examination Survey, characterizing a nationally representative sample of US adults aged ≥ 20 years.

**Results:**

A list of underlying conditions associated with high risk of severe RSV, influenza, or COVID-19 was generated from existing published sources (**Table 1**). Findings from our analysis suggest that among the adult population in the US (N=238,737,539), 53.6%, 23.8%, and 10.1% were estimated to have ≥ 1, ≥ 2, and ≥ 3 of the underlying conditions, respectively. The proportion of adults with ≥ 3 of the underlying conditions increased from 1.7% among those aged 20-49 years to 39.4% among those aged ≥ 80 years. Across all ages, 5.7% of Mexican American/Other Hispanic, 11.4% of non-Hispanic White, 11.4% of non-Hispanic Black, 5.3% of non-Hispanic Asian, and 11.1% of Other Race (including multiple races) individuals had ≥ 3 of the underlying conditions. Older-aged non-Hispanic Black individuals had higher prevalence of the underlying conditions relative to other races, ethnicities, and age groups (**Figure 1**). Notably, the proportion of non-Hispanic Black adults with ≥ 3 of the underlying conditions increased from 2.0% among those aged 20-49 years to 54.1% in those aged ≥ 80 years.

**Figure:**
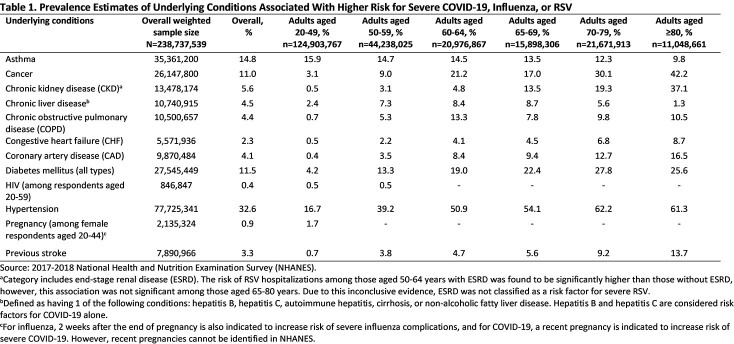

**Figure:**
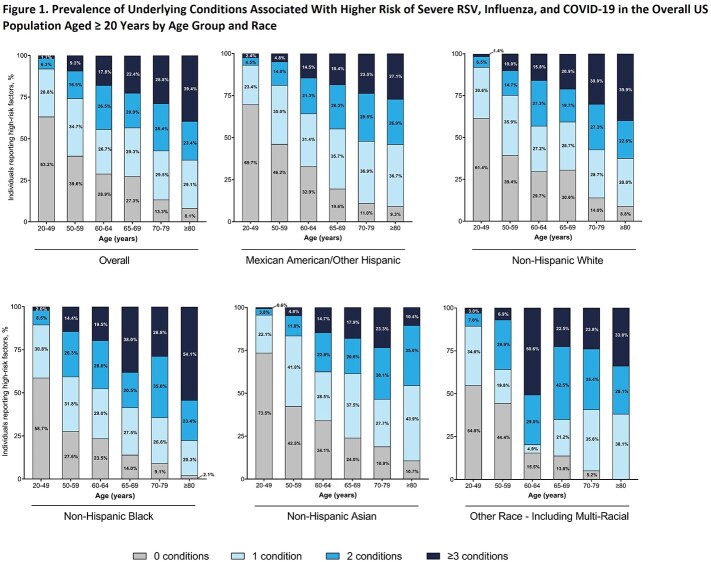

**Conclusion:**

These findings suggest that a substantial portion of the adult US population is at risk for severe RSV, influenza, or COVID-19, given their underlying health conditions. The prevalence of underlying conditions appears highest for older adults and non-Hispanic Black individuals. Our results suggest a need for preventive interventions against these respiratory viruses.

**Disclosures:**

**Parinaz Ghaswalla, PhD**, Moderna, Inc: Employee|Moderna, Inc: Stocks/Bonds **Abby Hitchens, MPH**, RTI Health Solutions (contracted by Moderna, Inc): Employee of RTI Health Solutions and was contracted by Moderna, Inc., to conduct this study **Sean D. Candrilli, PhD**, RTI Health Solutions (contracted by Moderna, Inc): Employee of RTI Health Solutions and was contracted by Moderna, Inc., to conduct this study. **Justin Carrico, BS**, RTI Health Solutions (contracted by Moderna, Inc): Employee of RTI Health Solutions and was contracted by Moderna, Inc., to conduct this study. **Katherine A. Hicks, MS**, RTI Health Solutions (contracted by Moderna, Inc): Employee of RTI Health Solutions and was contracted by Moderna, Inc., to conduct this study. **Eleanor Wilson, MD, MHS**, Moderna, Inc.: Employee|Moderna, Inc.: Stocks/Bonds **Darshan Mehta, PhD**, Moderna, Inc.: Employee|Moderna, Inc.: Stocks/Bonds **Catherine A. Panozzo, PhD**, Moderna, Inc.: Employee|Moderna, Inc.: Stocks/Bonds

